# Effect of a board game on imprisoned women’s knowledge about sexually transmitted infections: a quasi-experimental study

**DOI:** 10.1186/s12889-023-15646-3

**Published:** 2023-04-13

**Authors:** Isaiane da Silva Carvalho, Ryanne Carolynne Marques Gomes Mendes, Laís Helena de Souza Soares Lima, Gabrielle Pessôa da Silva, Monique de Freitas Gonçalves Lima, Tatiane Gomes Guedes, Francisca Márcia Pereira Linhares

**Affiliations:** Graduate Program in Nursing, Federal University of Pernambuco, Recife, PE Brazil

**Keywords:** Women, Prisons, Education in health, Educational technology, Sexually transmitted diseases

## Abstract

**Introduction:**

Board games can be used as a playful educational practice in the teaching and learning process, as they constitute an educational technology that can be a source of health knowledge and an aid in decision-making. The objective of this research was to assess the effect of a board game on imprisoned women’s knowledge about STIs.

**Methods:**

A quasi-experimental study was conducted in 2022 with 64 imprisoned women who were students at a school located in a prison unit from the city of Recife, state of Pernambuco, Brazil. A 32-item instrument was used to assess knowledge about sexually transmitted infections before, immediately after the intervention and at 15 days. The intervention consisted in applying the Previna board game in a classroom. All the analyses were performed in the Stata software, version 16.0, with a 5% significance level.

**Results:**

The knowledge mean in the pre-test was 23.62 (± 3.23) points, whereas it rose to 27.93 (± 2,28) in the immediate post-test, dropping to 27.34 (± 2.37) (p < 0.001) in post-test 2, which was performed 15 days after the intervention. There was a statistically significant difference in the means obtained between the pre-test and the immediate post-test (p < 0.001), with a difference of 4.241 points, as well as between the pre-test and post-test 2 (p < 0.001), a difference of 3.846 spots.

**Conclusions:**

The Previna board game significantly increased its players’ knowledge about STIs, and such increase in knowledge remained significant during the follow-up period.

## Background

Brazil has the fourth largest prison population in the world and the sixth highest rate of inmates for every 100,000 inhabitants. According to the National Penitentiary Information Survey (INFOPEN) in 2017, the Brazilian prison population was 726,354 inmates [1]. Of them, nearly 42,000 were women [2].

There are not enough prison units in the country, which results in overcrowding and in repercussions on the inmates’ quality of life and health [1]. It is noted that living in the prison system imposes greater exposure to physical risks, such as for the transmission of sexually transmissible infections (STIs) [3].

When compared to the general population, imprisoned women are more susceptible to contracting STIs. This can be explained by the lack of knowledge on the subject, distorted perceptions and risky behaviors adopted during incarceration. [4]. Thus, the female population tends to need more health care, mainly with regard to STI prevention, which is emphasized by the National Plan for Health in the Penitentiary System (*Plano Nacional de Saúde no Sistema Penitenciário*, PNSSP), which provides for health education, STI diagnosis, control and treatment actions; distribution of condoms; and elaboration of instructional educational material [3].

Health education actions and the development of instructional materials can be implemented through the use of educational technologies, which can support STI prevention and control. Educational games stand out among these technologies, which can be easily used by the prison population [1].

Educational games such as board games have been widely employed, as they enable better learning, clarification of doubts, acquisition of information and socialization. Board games can be used as a playful educational practice in the teaching and learning process, as they represent a source of health knowledge and an aid in decision-making. This type of game enables dialogue and favors reflection, allowing for better adherence to the practices to prevent diseases, such as STIs [5].

A systematic review evidences that board games exert positive impacts on knowledge about STIs and health-related behaviors [6], with the need to evaluate the effect of these games on knowledge in the female prison population [7]. This population has a history of problems related to sexuality and health [7], such as STIs, cervical cancer and unwanted pregnancies [8–10]. This assessment aims at contributing to preventing dissemination of STIs and their consequences.

Given the above, it is noted that transmission of diverse information related to sexual health is fundamental for STI prevention and control, as well as that educational games are mediating strategies for the construction of knowledge that can contribute to reducing STI rates among the prison population. In addition, the high STI rates in the female prison population and their consequences reinforce the need to work on the topic of STIs, in order to contribute to decision-making with a focus on harm reduction. The objective of this research is to assess the effect of a board game on imprisoned women’s knowledge about STIs.

## Methods

### Study design

A quasi-experimental study was developed in 2022 with 64 imprisoned women who were students at a school located in a prison unit in the City of Recife, State of Pernambuco, Brazil. The protocol for this study was published in an open access journal [11]. Intentionally, 7 classes out of a total of 10 were selected, so as to represent all the school modules, namely: I, III, V, VII, and 1st and 3rd year. The three excluded classes corresponded to modules contemplated in other shifts. As a safety measure for the research team, preference was given to daytime classes, when there was more than one class representing the same module. In the case of Module VII, classes from the afternoon and night shifts were included due to the reduced number of students found. Modules last for 6 months and correspond to school years outside prison.

Prior to this study, a study was carried out for the construction and validation of the data collection instrument and the board game. In both situations, the inmates participated in the semantic evaluation phase. Women who participated in these steps were not included in this study.

### Participants

In order to determine sample size, a sample calculation equation for the study of the mean in a single paired group was used [12], where: Z_(α/2)_ is Quantile of the standard normal (95% confidence, z = 1.96); Z_(1−β)_ is the Quantile of the standard normal for a test power of 80% (z = 0.84); σ_d_ = Expected standard deviation of the difference between the knowledge score before and after the intervention (σ_d_ = 2 points); and ∆ = Expected difference between the mean knowledge score before and after the intervention (∆ = µ_1_-µ_0_ = 8.5–7.4 = 1.1). The mean values were obtained in the pilot sample with 10 observations.

$$n = \frac{{\left( {2 \cdot \sigma _d^2} \right) \cdot {{\left( {{Z_{\alpha /2}} + {Z_{1 - \beta }}} \right)}^2}}}{{{\Delta ^2}}}$$

A 95% confidence level was adopted, as well as 80% test power. expected standard deviation of 2 points in the difference in the knowledge score before and after the intervention, and expected difference of 1.1 points between the mean knowledge score before and after the intervention. Based on these values, the required sample size was 53 participants. 20% was added to this number, considering eventual follow-up losses between baseline and 15 days after the intervention. Thus, the required sample size for the study corresponded to 64 women.

**Fig. 1:**
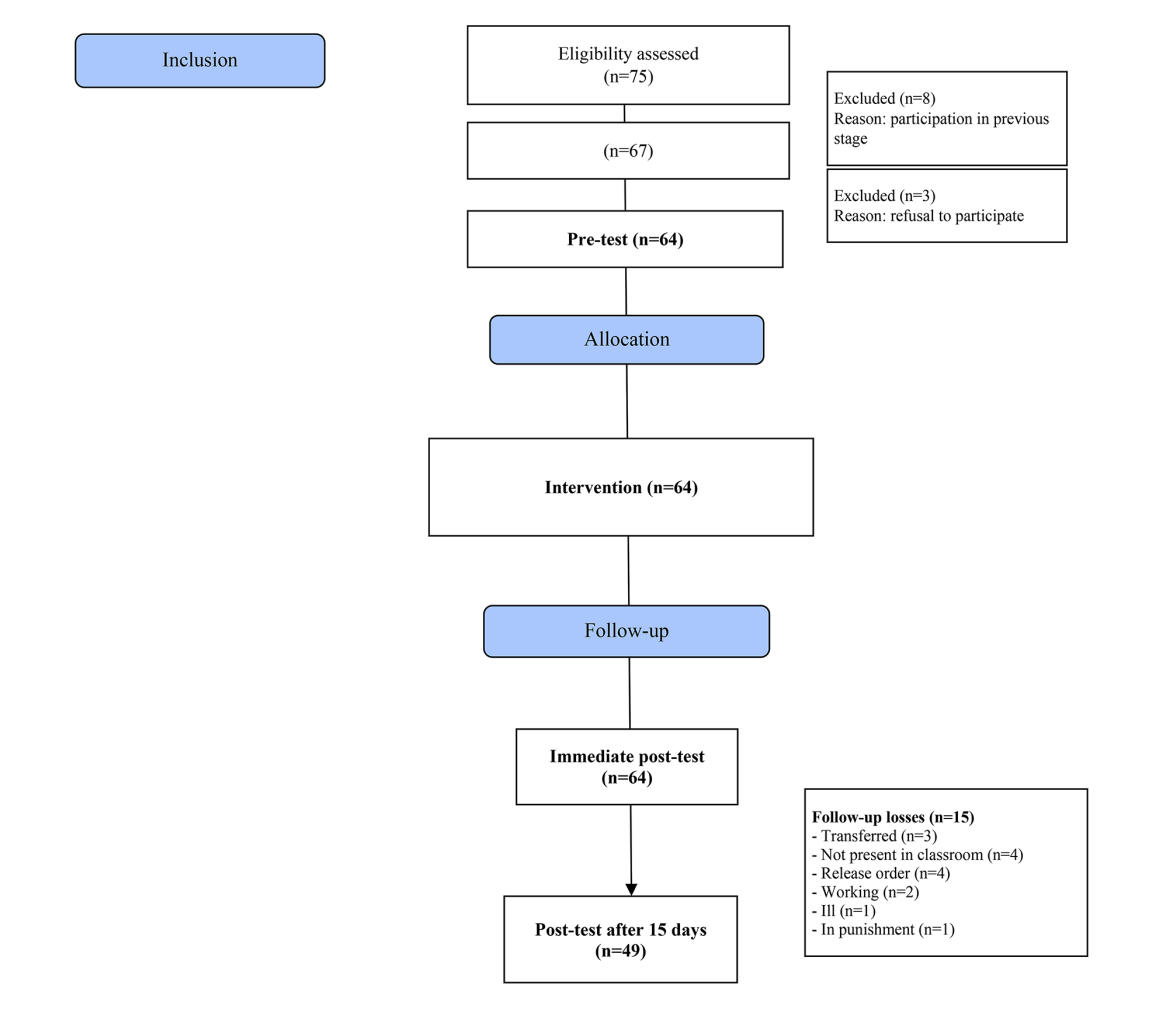
Follow-up flow corresponding to the participants

The eligibility or inclusion criteria were as follows: imprisoned women, regularly enrolled at the Olga Benário Prestes State School, aged 18 years old or over, literate, and expected to stay at least 15 days in the prison unit; in turn, the exclusion criteria were the following: women who participated in the semantic evaluation stages of the instrument or board game; and pregnant women, for having access to diverse information about STIs during prenatal consultations, which can be considered a bias; and the withdrawal or loss criteria corresponded to women who dropped out of the course (school evasion) after data collection was initiated, who had their deprivation of freedom suspended or ended, who were transferred to another prison unit, or who died. The process diagram corresponding to the study phases is presented in Fig. 1.

### Instruments

In order to assess the imprisoned women’s knowledge about STIs before and after the intervention, an instrument was used in two parts: (1) Characterization of the sample (social, demographic and prison situation); and (2) Knowledge about STIs. The instrument had 32 items and underwent a content validation process with health professionals, obtaining overall Content Validity Coefficient (CVC) values of 0.948 and 0.936 for representativeness and clarity, respectively. In addition to that, the semantic evaluation was performed with 10 imprisoned women, who considered the items as clear (Positivity Index = 99.0). The answer options were as follows: “Right”, “Wrong” and “I don’t know”. The correct answers received a score of “1” and the incorrect ones (Wrong/I don’t know) were scored with “0”. At the end, knowledge was assessed based on the score obtained, which varied from 0 to 32 points.

The instrument has elaborated based on the Clinical Protocol and Therapeutic Guidelines for the Comprehensive Care of People with Sexually Transmitted Infections in Brazil. The items refer to the following aspects: (a) signs and symptoms (5 items): myth about people with STIs; locatios the appearance of signs STIs; and main signs (wounds, discharges, and warts); (b) transmission (11 items): sex without a condom; sex between women; myth about using the bathroom; tattoo; piercing; sex toys; nail clippers; sharing syringes and needles; kiss on the mouth; gestation; It is part; (c) prevention (4 items): wearing a shirt; condom in oral sex; condom in anal sex; and vaccine against Hepatitis B; (d) main STIs (6 items): difference between HIV and AIDS; AIDS cure; cure of syphilis; HPV and cervical cancer; gonorrhea and pregnancy; and cure of Herpes; (e) treatment (5 items): search for a health professional; communication of partner; STIs treatment; treatment and use of the sweater; and partner treatment; (f) Vulnerability (1 item): imprisoned women compared to free women.

### Game board

**Fig. 2:**
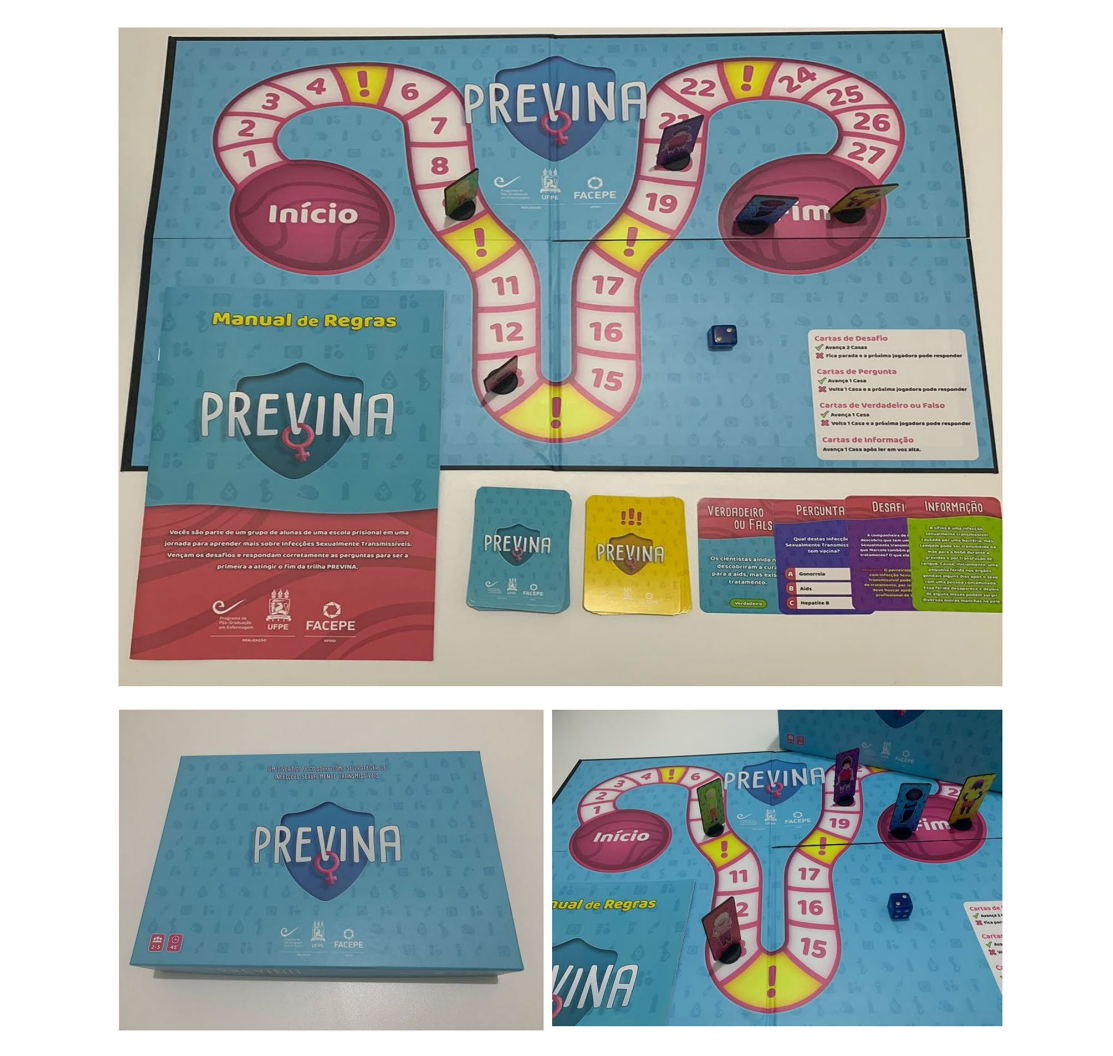
Previna board game

The educational intervention was conducted by applying the Previna board game, consisting of the following: 1 board; 1 instructions manual; 5 pawns; 52 cards; and 1 dice (Fig. 2). The board game underwent a content validation process in charge of 18 health and education professionals, obtaining an overall CVC of 0.966. In the board game content validation procedure regarding its appearance, which was conducted by 5 game designers/developers, the CVC was 0.917. All 10 imprisoned women who took part in the semantic evaluation asserted that they enhanced their knowledge, improved their motivation towards the class, and that they would like to play another game.

The educational technology was planned to be played with a minimum of 2 and a maximum of 5 players. The participants had the opportunity to play a single round of the game during the intervention. The classes were divided into small groups of a maximum of 5 students. For example, a class of 14 students was divided into 3 groups: 2 groups of 5 students and 1 group of 4 students.

The content on STIs – signs and symptoms, transmission, prevention, main STIs, diagnosis, treatment and vulnerability – was addressed based on situations that refer to the imprisoned women’s everyday life. The STIs addressed were: human immunodeficiency virus, syphilis, genital herpes, viral hepatitis, gonorrhea, chlamydia and human papillomavirus.

Different types of questions were posed to make the game more attractive, with different levels of difficulty. In addition, a type of information card was created that does not correspond to a question, but to a reading activity that must be carried out by the players. For example: (1) Multiple choice card: Maria found out she was pregnant in prison. One of the tests showed that she has syphilis. Can she have the treatment even though she is pregnant? Yes or No; (2) Challenge card: Marcela’s girlfriend found out that she has a sexually transmitted infection. Will Marcela also need treatment? What is she supposed to do?; (3) Prevention card: You performed all consultations and exams during prenatal care; (4) Risk card: You had your last cervical cancer screening more than 5 years ago.

### Interventions

The educational intervention was developed during June 2022 and the research team was divided as follows: all the interviews were conducted by the research assistants. The knowledge instrument was applied at 03 moments: T1 (initial interview), T2 (immediately after the intervention) and T3 (15 days after the intervention). The game was applied by the researcher in charge with the support of 2 research assistants. Data collection took place in three stages:

Stage 1: after selecting the women, they were invited to take part in the research. On this occasion, they were provided guidelines about the research objective and the procedures adopted for data collection. When a woman accepted, the Free and Informed Consent Form was read and signed. Subsequently, they were invited to participate in the structured individual interview to answer questions from the data collection instrument.

Stage 2: the intervention proposed for this study consisted in using the Previna board game in a classroom. The game was applied after the initial interview to groups with a maximum of 05 students. It is noted that all the women who were present in the classroom and volunteered to participate underwent the intervention. Immediately after the intervention, the STI knowledge instrument was reapplied.

Stage 3: the STI knowledge instrument was reapplied 15 days after the intervention. This application was individual and was performed in a classroom with the support of 5 research assistants. Once data collection has ended, the questions from the STI knowledge instrument were answered and discussed in the classroom, with a debate of the items that generated doubts.

### Statistical analysis

The data treatment and descriptive analysis procedures were performed by characterizing diverse sociodemographic information and data referring to the prison situation. Absolute and percentage distributions were calculated for the qualitative variables and position (mean and median) and dispersion (standard deviation) measures were calculated for the quantitative variables. The knowledge assessment instrument underwent a descriptive analysis of each question, separately, and of the total sum, in each of the three moments of its application: pre-test, immediate post-test and post-test 2. Both for each question and for the sum, position (mean and median) and dispersion (standard deviation) measures were calculated. The distribution corresponding to the representative variables of the sum of scores at each application moment was verified by means of the Shapiro-Wilk test. Friedman’s test was employed to compare the mean values obtained across all three application moments. In turn, the sum presented normal distribution in each of the periods analyzed; therefore, the difference of means analysis was performed via the ANOVA test for repeated samples. The Tukey HSD post-hoc test was used to identify the differences, as well as their significance, across all moments when the instrument was applied. All the analyses were performed in the Stata software, version 16.0, with a 5% significance level.

## Results

A total of 64 imprisoned women participated in the stage for evaluating the knowledge about STIs, with inclusion of participants from all six modules of the school. The social and demographic characterization data can be seen in Table 1. In terms of prison situation, 42 (65.63%) were arrested once, 18 (28.13%) had been convicted twice, and 4 (6.25%) had been convicted three times. For those who had been convicted more than once, the mean was 2.18 (± 0.39) times, varying from 2 to 3. The sum of the mean time of all the convictions was 24.07 (± 33.78) months, varying from 2 to 192 months, and the mean time corresponding to the current conviction was 15.82 (± 17.01) months, varying from 2 to 84. Most of the participants were awaiting trial (82.81%) and, in the case of those who had already been tried, the mean sentence was 14.04 (± 9.22) years, varying from 2 to 33. A total of 42 (65.63%) women stated having visits, especially from their mother 8 (19.05%) and children 7 (16.67%). Only 6 (9.38%) women worked in cooking or cleaning activities at the prison unit.

**Table 1:** Social and demographic characterization of the participants

Variable	n	%
**Marital status (n = 64)**		
Married/With a partner	18	28.13
Single	42	65.63
Separated/Divorced	1	1.56
Widow	3	4.69
**Number of children (n = 64)**		
None	10	15.63
One	11	17.19
Two	17	26.56
Three	13	20.31
Four and +	13	20.31
**Who do the children live with (n = 53)**		
Grandparents	25	47.17
Father	7	13.21
Uncles/Aunts	5	9.43
Other family members	5	9.43
Shelter	1	1.89
Independent	10	18.87
**Race (n = 64)**		
Brown	46	71.88
White	12	18.75
Black	4	6.25
Asian	2	3.13
**Religion (n = 63)**		
Evangelical	27	42.86
Catholic	18	28.57
Other	2	3.17
No religion	16	25.40
**She worked before being convicted (n = 62)**		
Yes	39	62.90
No	23	37.10
**Dependents receiving reclusion aid (n = 63)**		
No	63	98.43

When analyzing the means of each item, it was identified that the highest mean in the pre-test was obtained in item 27 (“In case of a sexually transmitted infection, you should seek the health professional of the prison unit”), Mean = 1, that is, it was answered correctly by all the participants. In turn, the worst result was in item 7 (“It is possible to catch sexually transmitted infections when using a bathroom shared by a lot of people”): Mean = 0.01 (± 0.12). In the immediate post-test, 6 items (5 - “A person can catch a sexually transmitted infection having sex without a condom”; 11 - “Condom use is the main way to prevent sexually transmitted infections”; 20 - “There is a vaccine to prevent Hepatitis B”; 27 - “In case of a sexually transmitted infection, the prison unit health professional should be sought”; 28 - “Any person who has a sexually transmitted infection must notify the partner”; and 31 - “The partner of a person with a sexually transmitted infection may need treatment”) obtained Mean = 1. The worst item was 26 (“Herpes has a cure”): Mean = 0.37 (± 0.48). In turn, in post-test 2, items 11, 27, 28 and 31 remained with correct answers for all the participants, and item 26 remained as the one with the worst result. In terms of difference in answers present in all three response times for each item, it is possible to observe that mean values with statistically significant differences were found (p < 0.05) for 13 of the 32 items of the instrument (2, 6, 7, 9, 14, 15, 19, 20, 21, 22, 23, 24 and 25), with increased mean values in all cases, when compared to the result obtained in the pre-test. Referring to the sum of the items, a mean of 23.62 (± 3.23) points was identified in the pre-test. In turn, in the immediate post-test, the mean rose to 27.93 (± 2.28) and dropped to 27.34(± 2.37) in post-test 2, representing a statistically significant difference (p < 0.001) (Table 2).

**Table 2:** Knowledge about Sexually Transmitted Infections in the pre-test, immediate post-test and post-test 2

Item No.	Pre-test (n = 64)		Immediate post-test (n = 64)		Post-test 2 (n = 49)		p-value
	Mean (± SD)	Median (IQR)	Mean (± SD)	Median (IQR)	Mean (± SD)	Median (IQR)	
1. A seemingly healthy person may have some Sexually Transmitted Infection	0.85 (0.30)	1 (1)	0.92 (0.27)	1 (1)	0.95 (0.19)	1 (0)	0.152^‡^
2. The main signs of Sexually Transmitted Infections appear in the private parts	0.81(0.31)	1 (1)	0.98 (0.12)	1 (0)	0.91 (0.27)	1 (0)	0.002^‡^
3. Mouth sores can also be a sign of Sexually Transmitted Infections	0.82(0.38)	1 (1)	0.92 (0.27)	1 (1)	0.91 (0.27)	1 (0)	0.176^‡^
4. Discharges in the private parts can be a sign of Sexually Transmitted Infections	0.85 (0.35)	1 (1)	0.92 (0.27)	1 (1)	0.91 (0.27)	1 (0)	0.433^‡^
5. A person can catch a Sexually Transmitted Infection by having sex without a condom	1.0 (0)	1 (0)	1.0 (0)	1 (0)	0.97 (0.14)	1 (0)	0.310^‡^
6. A woman can catch a Sexually Transmitted Infection by having sex with another woman	0.78 (0.41)	1 (1)	0.93 (0.24)	1 (1)	0.95 (0.19)	1 (0)	0.003^‡^
7. It is possible to catch Sexually Transmitted Infections when using a bathroom shared by a lot of people	0.01 (0.12)	0 (0)	0.56 (0.50)	1 (1)	0.51 (0.50)	1 (1)	< 0.001^‡^
8. It is possible to catch Sexually Transmitted Infections by getting a tattoo	0.90 (0.29)	1 (1)	0.98 (0.12)	1 (0)	0.97 (0.14)	1 (0)	0.065^‡^
9. It is possible to catch Sexually Transmitted Infections by wearing a piercing	0.78 (0.41)	1 (1)	0.96 (0.17)	1 (0)	0.91 (0.21)	1 (1)	0.001^‡^
10. It is possible to catch Sexually Transmitted Infections by sharing sex toys (e.g., vibrators)	0.89 (0.31)	1 (1)	0.95 (0.21)	1 (0)	0.97 (0.14)	1 (0)	0.117^‡^
11. Condoms are the main way to prevent Sexually Transmitted Infections	0.95 (0.21)	1 (0)	1.0 (0)	1 (0)	1.0 (0)	1 (0)	0.068^‡^
12. Condoms can be used in oral sex to prevent Sexually Transmitted Infections	0.89 (0.31)	1 (1)	0.96 (0.17)	1 (0)	0.91 (0.27)	1 (0)	0.269^‡^
13. Condoms can be used in anal sex to prevent Sexually Transmitted Infections	0.89 (0.31)	1 (1)	0.98 (0.12)	1 (0)	0.97 (0.14)	1 (0)	0.029^‡^
14. It is possible to get HIV when giving a kiss on the mouth	0.29 (0.46)	0 (1)	0.50 (0.50)	0.50 (1)	0.53 (0.50)	1 (0)	0.011^‡^
15. Having HIV is the same as having AIDS.	0.25 (0.43)	0 (1)	0.50 (0.50)	0.50 (1)	0.40 (0.49)	0 (1)	0.009^‡^
16. There is a cure for AIDS	0.81 (0.39)	1 (1)	0.81 (0.39)	1 (1)	0.81 (0.39)	1 (1)	0.919^‡^
17. Syphilis can be passed on from mother to baby during pregnancy	0.89 (0.31)	1 (1)	0.95 (0.21)	1 (0)	0.93 (0.24)	1 (0)	0.385^‡^
18. Syphilis has a cure	0.75 (0.43)	1 (1)	0.82 (0.38)	1 (1)	0.79 (0.40)	1 (1)	0.367^‡^
19. Hepatitis B can be transmitted when sharing syringes and needles	0.82 (0.38)	1 (1)	0.95 (0.21)	1 (0)	0.95 (0.19)	1 (0)	0.017^‡^
20. There is a vaccine to prevent hepatitis B	0.85 (0.35)	1 (1)	1.0 (0)	1 (0)	0.87 (0.33)	1 (1)	0.013^‡^
21. Hepatitis C can be transmitted when sharing nail pliers	0.76 (0.42)	1 (1)	0.95 (0.21)	1 (0)	0.97 (0.14)	1 (0)	< 0.001^‡^
22. Warts on the private parts are caused by HPV	0.54 (0.50)	1 (1)	0.87 (0.33)	1 (1)	0.77 (0.42)	1 (1)	< 0.001^‡^
23. Cervical cancer can be caused by HPV	0.65 (0.47)	1 (1)	0.84 (0.36)	1 (1)	0.81 (0.39)	1 (1)	0.029^‡^
24. A woman with gonorrhea may have trouble getting pregnant	0.50 (0.50)	0.50 (1)	0.87 (0.33)	1 (1)	0.79 (0.40)	1 (1)	< 0.001^‡^
25. Chlamydia can be passed on from mother to baby during delivery	0.50 (0.50)	0.50 (1)	0.76 (0.42)	1 (1)	0.74 (0.43)	1 (1)	0.001^‡^
26. Herpes has a cure	0.20 (0.40)	0 (1)	0.37 (0.48)	0 (1)	0.34 (0.48)	0 (1)	0.082^‡^
27. In case of a Sexually Transmitted Infection, the health professional working in the prison unit should be sought	1.0 (0)	1 (0)	1.0 (0)	1 (0)	1.0 (0)	1 (0)	-
28. Any person with a Sexually Transmitted Infection should notify their partner	0.98 (0.12)	1 (0)	1.0 (0)	1 (0)	1.0 (0)	1 (0)	0.413^‡^
29. There is treatment for Sexually Transmitted Infections	0.95 (0.21)	1 (0)	0.98 (0.12)	1 (0)	0.95 (0.19)	1 (0)	0.640^‡^
30. A person undergoing treatment for a Sexually Transmitted Infection can have sex without a condom	0.87 (0.33)	1 (1)	0.90 (0.29)	1 (1)	0.97 (0.14)	1 (0)	0.107^‡^
31. The partner of a person with some Sexually Transmitted Infection may need treatment	0.95 (0.21)	1 (0)	1.0 (0)	1 (0)	1.0 (0)	1 (0)	0.068^‡^
32. A woman in the prison unit is at an increased risk of having a Sexually Transmitted Infection than a free woman	0.53 (0.50)	1 (1)	0.70 (0.46)	1 (1)	0.67 (0.47)	1 (1)	0.106^II^
**Sum**	**23.62 (3.23)**	**24 (10)**	**27.93 (2.28)**	**28 (5)**	**27.34 (2.37)**	**28 (6)**	**< 0.001** ^**II**^

^‡^ Friedman test. ^II^ ANOVA for repeated samples

Table 3 describes the differences of means and their respective significance values when comparing all 3 moments. It can be identified that there was a statistically significant difference in the mean values obtained between the pre-test and the immediate post-test (p < 0.001), with a difference of 4.241 points. Likewise, there was also a difference between the pre-test and post-test 2 (p < 0.001): 3.846 points. On the other hand, the difference of 0.394 points between the immediate post-test and post-test 2 results was not statistically significant (p > 0.05).

**Table 3:** Comparisons of the sum referring to knowledge about Sexually Transmitted Infections in the pre-test, immediate post-test and post-test 2

Moment	Immediate post-test		Post-test 2	
	Difference	p-value^II^	Difference	p-value^II^
**Post-test 2**	-0.394	1.00	-	-
**Pre-test**	-4.241	< 0.001	-3.846	< 0.001

^**II**^ Tukey HSD post-hoc test

## Discussion

The Previna board game was designed to be a complementary tool in the teaching and learning process about STIs for imprisoned women. The results showed that this educational technology significantly increased the players’ knowledge about STIs and that this increase in knowledge remained significant during follow-up. This suggests that there was knowledge retention in the period researched. The increase in knowledge was also present in other studies that used board games for educational purposes [13–16].

The knowledge retention observed in this study is a result that draws attention, as the school teachers frequently mentioned that the women had difficulty retaining the contents taught in the classroom. This suggests that the approach to content may or may not favor this process. In this way, the board game can be used to present educational content to adult students in order to address their learning needs [14] and to make learning more significant and engaging.

Regardless of the teaching method chosen, the teacher must employ strategies during the classes that are capable of increasing the student’s motivation, improving knowledge retention and learning results [16]. It is known that, in the prison environment, the system itself limits some actions. Thus, it is increasingly important to use accessible technologies that may streamline the knowledge construction process and make it more active.

The increase in knowledge remained significant after 15 days, suggesting short-term knowledge gain. Previous studies have also evidenced short-term knowledge gains through learning using games [14, 17–19].

It is important to highlight that the knowledge mean in the pre-test (23.62; SD = 3.23) was expressive since, from a total of 32 items present in the instrument, approximately 74% were answered correctly. Such result shows that there is certain level of knowledge on the subject matter in the sample under study. A similar result was found by a study that evaluated knowledge about HIV and the Hepatitis C Virus (HCV) among imprisoned North American women and identified high levels of knowledge about HIV and HCV, despite extensive reports of risk behaviors as sex work (44%) and history of injecting drug use (75.5%). [20].

This can be the result of educational actions developed in a previous period, such as the extension project entitled “Sexual and Reproductive Health Care for women in custody”, carried out in the prison unit in partnership with the Nursing Department of the Federal University of Pernambuco, started in 2013 [21] and which had its activities interrupted due to the COVID-19 pandemic in 2020.

In addition, the imprisoned women in question were students at a prison school and the content on STIs may have been worked on in a previous period and in a transversal way, as part of YAE activities and within the scope of knowledge areas such as Natural Sciences, as provided by the Common National Curriculum Base [22, 23]. However, if the game was able to increase knowledge about STIs in this context, it is possible that it will present more expressive results if the baseline knowledge levels are lower.

In relation to the item that presented the worst performance in the pre-test (item 7) and which deals with STI contagion through the use of shared bathrooms, it is noticed that this is a myth widely spread in our society. Some papers also evidenced this view by parents and teachers in Kenya [24], by seafarers in Montenegro [25] and about a possible toilet disease, with signs and symptoms similar to STIs by young Nigerian women, who believed they acquired the disease from using a toilet with poor hygiene conditions [26].

In addition to that, a study on the perspectives of risk factors for STIs by Brazilian imprisoned women evidenced that sexual behavior and unprotected sex were not understood as the main ways of acquiring STIs in this environment, but sharing the bathroom as a risk potentiator [2]. In view of this, it is important to reinforce the ways in which STIs are transmitted among this population group, which does not mean saying that hygiene care in these places should be neglected. It is also noted that there was a significant increase in the means for this item after the intervention.

Item 26, which deals with the existence of a cure for herpes and corresponds to a false item, presented the worst performance among the items, both in the immediate post-test and in post-test 2, and the increase in means was not significant, although it was an STI addressed in the board game through 4 cards. This can be the result of unfamiliarity with this STI by women and that it is not so publicized. A study on knowledge about STIs in a male imprisoned population showed that the interviewees had difficulty identifying diseases such as genital herpes [27]. Thus, it is important to think of strategies to broaden discussions about this disease in order to increase knowledge on the subject matter, mainly because, despite the existence of antivirals for treatment, there is still no cure [28].

A total of 13 items presented statistically significant differences. However, depending on the number of analyses, it should be noted that some of these findings may be due to type I error. Item 2, which deals with the main signs of STIs appearing in the intimate parts, despite already obtaining a baseline mean above 0.8, managed to significantly increase the number of correct answers after the intervention. It is important to note that the game, as well as the moment when the questions were discussed at the end of data collection, reinforced the understanding that this was not the only place where signs of STIs could appear, but that such areas frequently present some alteration due to the infections [29, 30].

Items 6 and 9, which deal with STI transmission by women who have sex with women and through piercing, also presented statistically significant differences. On the subject matter, a Brazilian research study on vulnerability to STIs among women who have sex with women showed lack of risk perception for STIs (56.7%) and HIV (67.3%) [31] by this group.

In addition to that, in the prison environment, some practices such as body modifications resulting from tattoos and piercings can increase the risk of viral hepatitis infection [32]. Thus, lack of knowledge on the subject matter can increase sexual risk behaviors and promote neglect of individual care measures aimed at STI prevention.

Items 14 and 15, which addressed HIV transmission through kissing on the mouth and the difference between HIV and AIDS, initially with means below 0.30, also presented statistically significant differences. During the game, the ways in which HIV is transmitted and the difference between HIV infection and AIDS stood out. A study conducted with aged Brazilians also identified that 38.1% believed that kissing on the mouth was a means of HIV transmission [33]. Likewise, the conception that HIV and AIDS were the same thing was also found among Brazilian adolescents [34].

Items 19, 20 and 21, which deal with Hepatitis B and C transmission through syringes, needles and nail pliers, as well as with the existence of a vaccine against Hepatitis B, started with means above 0.75, suggesting knowledge about the subject matter, and rose significantly during follow-up. An important result, considering that the use of syringes, needles and nail pliers is a constant factor in this environment [35, 36]. Likewise, being aware of the Hepatitis B vaccine is the first step in preventing this infection, which has an increased risk of affecting the prison population [37].

In relation to items 22 and 23, which deal with HPV and the occurrence of genital warts and cervical cancer, respectively, it was observed that the initial means were lower. Slightly more than half of the interviewees gave correct answers to the items in the pre-test phase. Such fact can be the result of knowledge gaps about the consequences of HPV infection. A study with imprisoned American women identified that 42% and 57% gave correct answers to the questions about HPV causing genital warts and cervical cancer, respectively [38]. During the game, the cards about HPV infection may have contributed to the results obtained in the follow-up, as the increases were statistically significant.

Finally, items 24 and 25, which deal with gonorrhea and chlamydia infections and that also showed statistically significant differences, with improved results, it was noticed that both items presented an initial knowledge mean of 0.5. A study that evaluated young Greeks’ knowledge about STIs identified that only 17.6% considered that gonorrhea can cause infertility [39]. It is known that these infections have serious consequences for women and also for their children [28, 40], being opportune that more frequent discussions take place to inform this population group about prevention and harm reduction strategies.

In terms of the items where no statistically significant differences were observed, it is noted that, of the total of 19 items under these conditions, 15 started with means above 0.8 in the pre-test, showing that there is already relative knowledge on the subject matter. Thus, the differences produced in the following measurements were insufficient to be considered significant. In addition to that, item 27, which deals with the need to seek professional help in case of an STI, was correctly answered by all women at all three measurement moments.

Contrary to this pattern, there are items 18, 26, already discussed, and 32. The first deals with the existence of a cure for syphilis. Despite an expressive mean, 0.75 in the pre-test, there were many doubts about the item. According to some women, in a previous period there was a lecture at the unit, which mentioned non-existence of a cure for syphilis, based on the premise that the exams would always be positive for a previously infected person, even if they had undergone adequate treatment. This leads to the belief that there was misunderstanding about the idea of a serological cure. This is defined as at least a 4-fold reduction in the treponemal titers or seroconversion to non-reagent results [41]. Thus, the presence of treponemal titers in the exam does not necessarily mean that the disease is present. The treatment can be considered successful when the aforementioned decline occurs, after using antibiotic therapy, in a period of 12 months for early syphilis and 24 months for late syphilis [42].

Item 32, which started with an even lower mean in the pre-test (0.53) and deals with imprisoned women’s vulnerability to STIs when compared to free women, was a subject matter of great controversy. Despite the game portraying this idea, based on scientific studies [4, 43, 44], it was common for imprisoned women to disagree with this information. This is because the unit provides access to a series of preventive services that, under conditions of freedom, they would have difficulty enjoying, taking into account the social vulnerability of the environment where they were previously inserted. This reinforces the idea that imprisonment can also be seen as a unique opportunity to work on health promotion and disease prevention with a population group that is naturally vulnerable in several aspects.

Finally, the Previna board game made it possible for imprisoned women to increase the mean of correct answers to the items present in the knowledge assessment instrument and, therefore, fulfilled its objective of increasing knowledge about STIs.

The fact that this is a quasi-experimental study does not allow making causal assertions about the board game. In addition to that, the evaluation of the board game effect only considered the short-term, not being possible to identify knowledge retention in the medium- and long-term; the imprisoned women were students at a prison school, which means that the data do not necessarily reflect the reality of incarcerated women as a whole; only the knowledge outcome was evaluated; and only one prison unit was analyzed, although it is the one with the largest number of imprisoned women in the state.

## Conclusion

The Previna board game had its effect tested through a quasi-experimental study that evidenced a significant increase in knowledge about STIs immediately after the intervention and also in the follow-up, 15 days later. It is recommended that future research resorts to more robust studies, such as randomized clinical trials, and compares the effect of the Previna Board Game with other educational technologies. In addition, it is important to consider new follow-up intervals to measure medium- and long-term knowledge retention, other outcomes such as the incidence of STIs, and imprisoned women who are not necessarily students in prison units. Finally, a qualitative work could be very pertinent to explore what the participants thought of the game and in trying to understand why the game was apparently effective in improving knowledge about some STIs, but not for others.

## Data Availability

The data generated and used in the analysis of this study are included in this article. The corresponding author will provide additional data upon request.

## References

[1] Carvalho FF, Takeda E, Chagas EFB (2020). Knowledge of the prison population about sexually transmitted infections. Rev Gaúcha de Enferm.

[2] Carvalho IA, Nodari PRG, Nascimento JA et al. Perspectives of incarcerated women on risk factors for sexually transmitted infection: exploratory and qualitative study. Enferm. Actual Costa Rica. 2021;(40):44056.

[3] Nichiata LYI, Martins NVN, Viana LV et al. Prevalence of sexually transmitted infections in women freedom of private. Revista Saúde (Sta. Maria); 2019;45(1).

[4] Benedetti MSG, Nogami ASA, Costa BB (2020). Sexually transmitted infections in women deprived of liberty in Roraima, Brazil. Rev Saude Publica.

[5] Martins AS, Silva ML, Amaral TF (2022). Playful-educational activities on sexually transmitted infections: scientific dissemination proposal in the school environment. Res Soc Dev.

[6] Gauthier A, Kato PM, Bul KCM (2019). Board games for health: a systematic literature review and meta-analysis. Games Health J.

[7] Geana MV, Anderson S, Lipnicky A (2021). Managing Technology, Content, and user experience: an mHealth intervention to Improve Women’s Health literacy after incarceration. J Health Care Poor Underserved.

[8] Peart MS, Knittel AK (2020). Contraception need and available services among incarcerated women in the United States: a systematic review. Contracept Reprod Med.

[9] Paynter M, Heggie C, McKibbon S (2022). Sexual and Reproductive Health Outcomes among Incarcerated Women in Canada: a scoping review. Can J Nurs Res.

[10] Kelly PJ, Hunter J, Daily EB (2017). Challenges to pap Smear follow-up among women in the Criminal Justice System. J Community Health.

[11] Carvalho IS, Mendes RCMG, Lima LHSS (2022). Effect of a board game about sexually transmitted infections on imprisoned women’s knowledge: protocol for a quasi-experimental study. BMJ Open.

[12] Arango HG (2011). Bioestatística: teórica e computacional com banco de dados reais em disco.

[13] Wanyama JN, Castelnuovo B, Robertson G (2012). A randomized controlled trial to evaluate the effectiveness of a Board game on patients’ knowledge uptake of HIV and sexually transmitted Diseases at the infectious Diseases Institute, Kampala, Uganda. J Acquir Immune Defic Syndr.

[14] Cutumisu M, Patel SD, Brown MRG et al. RETAIN: A Board Game That Improves Neonatal Resuscitation Knowledge Retention. Front Pediatr. 2019;7(13). 10.3389/fped.2019.00013 PMC6365420 30766862

[15] Liu HY, Chen PH, Chen WJ (2021). The effectiveness of a Board game-based oral Hygiene Education Program on oral Hygiene Knowledge and Plaque Index of adults with intellectual disability: a pilot study. Int J Environ Res Public Health.

[16] Chang YS, Hu SH, Kuo SW (2022). Effects of board game play on nursing students’ medication knowledge: a randomized controlled trial. Nurse Educ Pract.

[17] Martins FDP, Leal LP, Linhares FMP (2018). Effect of the board game as educational technology on schoolchildren’s knowledge on breastfeeding. Rev Latino-Am Enfermagem.

[18] Hu H, Lai X, Yan L (2021). Improving nursing students’ COVID-19 knowledge using a serious game. Comput Inf Nurs.

[19] Hu H, Lai X, Li H, Nyland J. Teaching Disaster Evacuation Management Education to Nursing Students Using Virtual Reality Mobile GameBased Learning. Comput Inform Nurs. 2022. 10.1097/CIN.0000000000000856 35485942

[20] Peteet B, Staton M, Miller-Roenigk B (2018). Rural incarcerated women: HIV/HCV Knowledge and Correlates of Risky Behavior. Health Educ Behav.

[21] Guedes TG, Linhares FMP, Morais SCRV (2015). Health education: strategy for sexual and reproductive care for women in custody. Procedia.

[22] Ministério da Educação do Brasil (2017). Base Nacional Comum Curricular.

[23] Dias DF, Sposito NEC (2021). Educação sexual: uma sequência didática para a EJA de uma escola de assentamento. Educ rev.

[24] Wanje G, Masese L, Avuvika E (2017). Parents’ and teachers’ views on sexual health education and screening for sexually transmitted infections among in-school adolescent girls in Kenya: a qualitative study. Reprod Health.

[25] Ljiljana J, Milan P, Jasmina S (2019). Awareness of HIV/AIDS and other sexually transmitted infections among the montenegrin seafarers. Vojnosanit Pregl.

[26] Oripelaye MM, Olasode OA (2015). The myth of Toilet Disease: an impediment to the control of sexually transmitted infection among young females. Nigerian J Dermatology.

[27] Carvalho IS, Guedes TG, Bezerra SMMS (2020). Educational technologies on sexually transmitted infections for incarcerated women. Rev Latino-Am Enfermagem.

[28] Tuddenham S, Hamill MM, Ghanem KG (2022). Diagnosis and treatment of sexually transmitted infections: a review. JAMA.

[29] Mccormack D, Koons K (2019). Sexually transmitted infections. Emerg Med Clin North Am.

[30] Roett MA (2020). Genital ulcers: Differential diagnosis and management. Am Fam Physician.

[31] Andrade J, Ignácio MAO, Freitas APF (2020). Vulnerability to sexually transmitted infections of women who have sex with women. Ciênc saúde coletiva.

[32] Parriault MC, Chaponnay A, Cropet C (2019). Penile implants and other high risk practices in french Guiana’s correctional facility: a cause for concern. PLoS ONE.

[33] Bastos LM, Tolentino JMS, Frota MAO (2018). Evaluation of the level of knowledge about Aids and syphilis among the elderly from a city in the interior of the state of Ceará. Brazil Ciênc saúde colet.

[34] Dossantos FNC, da Silva BCO, Barreto VP, Costa FH da, Medeiros R, de Feijão ER. AR. Implementation of peer education for HIV

[35] Pinho TAM, Silva AO, Pimenta CJL, Moreira MASP (2018). Social representations of imprisoned women on acquired Immunodeficiency Syndrome. Rev Rene.

[36] Moazen B, Stoven H, Dolan K (2020). Prisoners should not be left behind in HCV research and policies. Harm Reduct J.

[37] Stasi C, Monnini M, Cellesi V (2019). Screening for hepatitis B virus and accelerated vaccination schedule in prison: a pilot multicenter study. Vaccine.

[38] Moore A, Cox-Martin M, Dempsey AF (2019). HPV Vaccination in Correctional Care: knowledge, attitudes, and barriers among incarcerated women. J Correct Health Care.

[39] Voyiatzaki C, Venetikou NS, Papageorgiou E (2021). Awareness, knowledge and risky behaviors of sexually transmitted Diseases among Young People in Greece. Int J Environ Res Public Health.

[40] He W, Jin Y, Zhu H (2020). Effect of Chlamydia trachomatis on adverse pregnancy outcomes: a metaanalysis. Arch Gynecol Obstet.

[41] Atsawawaranunt K, Kittiyaowamarn R, Phonrat B (2020). Time to Serological Cure and Associated factors among Syphilis patients with and without HIV in a sexually transmitted Infections Center, Thailand. Sex Transm Dis.

[42] Workowski KA, Bachmann LH, Chan PA (2021). Sexually transmitted Infections Treatment Guidelines, 2021. MMWR Recomm Rep.

[43] Martins DC, Pesce GB, Silva GM, Fernandes CAM (2018). Sexual behavior and sexually transmitted diseases among the female partners of inmates. Rev Latino-Am Enfermagem.

[44] Lôbo MP, Penna LHG, Carinhanha JI (2019). Actions to prevent and cope with the STI/AIDS experienced by women in prison. Rev enferm UERJ.

